# Determinants of antenatal care service satisfaction among pregnant women at Kawaala Health Centre IV, Kampala City: a cross-sectional analytical study

**DOI:** 10.3389/frph.2026.1864242

**Published:** 2026-08-05

**Authors:** Elizabeth Nshabohurira, Eliot Namanya, Ronald Omolo Ouma, Eleanor Turyakira, Patience Amutuhaire, Scovia G. Mbabazi, Nathan Mugenyi, Pauline Byakika-Kibwika

**Affiliations:** 1 Faculty of Health Sciences, Uganda Martyrs University, Kampala, Uganda; 2 Department of Physiology, Faculty of Medicine, Mbarara University of Science and Technology, Mbarara, Uganda; 3 Department of Community Health, Faculty of Medicine, Mbarara University of Science and Technology, Mbarara, Uganda; 4 Department of Pharmacology and Therapeutics, Faculty of Medicine, Mbarara University of Science and Technology, Mbarara, Uganda; 5 Office of the Vice Chancellor, Mbarara University of Science and Technology, Mbarara, Uganda

**Keywords:** ANC satisfaction, antenatal care (ANC), Kawaala Health Centre IV, pregnant women, Uganda

## Abstract

**Background:**

Antenatal care (ANC) in developing countries is among strategies aimed at reducing maternal mortality yet its quality and utilization are still low. Only about 60% of pregnant mothers in Uganda attend at least four ANC visits despite it being one of the strategies to curtail maternal mortality. The satisfaction of pregnant mothers with ANC services is associated with increasing their access and utilization. Therefore, there is need to evaluate the extent to which pregnant mothers are satisfied with ANC services and the associated factors.

**Methods:**

A cross-sectional analytical study employing quantitative methods of data collection and analysis was conducted. Data was collected from 384 participants using interviewer administered semi structured questionnaires. Bivariate analysis using the Pearson chi-square (*χ*2) test was conducted to determine the individual and health facility factors associated with satisfaction with ANC services among pregnant women. Multivariate analysis was performed on the variables that were statistically significant (*p* < 0.05) at bivariate analysis using logistic regression model to control confounding and determine independent factors associated with satisfaction with ANC services among pregnant women.

**Results:**

Of the 384 pregnant women enrolled, 259 (67.4%) were satisfied with ANC services. Multivariable logistic regression analysis revealed that knowledge about ANC services (AOR=4.2, 95% CI: 2.23–7.81; *p* < 0.001), waiting time of less than one hour (AOR=2.8, 95% CI: 1.30–5.99; *p* = 0.009), positive attitude of healthcare providers (AOR=5.9, 95% CI: 3.13–11.25; *p* < 0.001), and availability of ANC commodities (AOR=3.0, 95% CI: 1.53–6.06; *p* = 0.002) were independently associated with satisfaction with ANC services.

**Conclusion:**

Improving maternal knowledge on ANC services, reducing waiting times, ensuring availability of essential ANC commodities, and promoting respectful provider-client interactions may enhance women's satisfaction with ANC services. Strengthening these aspects of care could improve ANC utilization and contribute to better maternal health outcomes.

## Introduction

Antenatal care (ANC) is routine care provided to a pregnant woman between conception up to the onset of labor (1). ANC is a crucial maternal and child health service, which facilitates early detection and management of pregnancy-related complications while offering health education, counselling, preventive therapies and referrals where necessary (2). Patient satisfaction, an important measure of health system responsiveness, has been shown to not only influence uptake and utilization of healthcare interventions, but also adherence to antenatal care (3). In Sub Saharan Africa (SSA), patient satisfaction has been linked to adherence to HIV/AIDS treatment and maternity care services (3).

In spite of progress in maternal and child health (MCH) programs, the global burden remains significant. Approximately, two thirds of maternal, newborn and child deaths could be prevented if mothers fully attend ANC services (4). Globally, close to 800,000 neonatal deaths and approximately 160,000 in SSA could be prevented through improved ANC coverage (4, 5). Nevertheless, maternal mortality continues to be unacceptably high. Every day in 2023, over 700 women died from preventable causes related to pregnancy and child birth, meaning that approximately one woman is dying every two minutes (6). In 2015, 303,000 women died from pregnancy and childbirth-related complications, with 99% (302,000) of them occurring in developing regions, and SSA alone accounted for about 66% (201,000) deaths (7).

Maternal mortality ratio remains high across the globe at 223 per 100,000 live births, 545 per 100,000 live births in the SSA, and 336 per 100,000 live births in Uganda (8, 9). Indicators of child survival also remain poor, with the global under-five mortality estimates at 37 deaths per 1000 live births (10), 62 deaths per 1000 live births in SSA (11) and 38.8 deaths per 1000 live births in Uganda (12).

Uganda had some improvements in ANC coverage between 2011 and 2016, where ANC coverage increased to 97% contributing to improvement of skilled birth attendance from 57% to 73% in the period (13). The proportion of infant deaths prevented by 11%, and child mortality reduced by 16% while the proportion of children under five years saved were 34% (13). However, while 90% to 94% of women attend at least the first ANC visit, only 65% of urban and 58% of rural women complete the recommended minimum of four visits (4). This gap is an indicator that service continuity is still a challenge, and maternal satisfaction with ANC services is a primary factor associated with ANC attendance.

Mother's satisfaction with ANC services is an important part of the quality assurance process in health systems, that offers a potential to improve the access to and utilization of the services. Mothers’ perceptions of antenatal visits significantly affect how they assess the quality of care, which in turn affects their utilization of the services. Patient satisfaction has occasionally been linked to the quality of services rendered and the extent to which individual needs are met. Satisfied patients are likely to come back for the services and recommend services to others, while dissatisfaction may discourage further attendance. Failure of pregnant mothers to attend ANC has negative impacts on the mother and unborn child and according to the World Health Organization (WHO), most cases of maternal and perinatal mortality occur in women who receive no antenatal care with more than 99% of these women living in developing countries.

The benefits of antenatal care are well documented (14, 15). In a study conducted in Ethiopia, postpartum hemorrhage, asphyxia and still births were more prevalent among mothers who did not complete four ANC visits compared to those mothers who did. In the same study, early neonatal death and low birth weights were reduced by 61.3% and 46.5% respectively when mothers attended ANC early in the first trimester (16). In addition, improved ANC attendance has been seen as a fulcrum to good child health, development and the genesis of equity to lifelong health and productivity of the mother and the family (17).

To maximize these benefits, WHO previously recommended four focused ANC visits, with the first visit occurring in the first trimester (18). However, recently WHO has increased the frequency of the ANC care contacts to eight with the first care contact happening within the first trimester of gestation and subsequent visits expected four weeks apart (19). In spite of these global recommendations, the 2016 Ugandan Demographic Health Survey (UDHS) reported that only 60% of the pregnant mothers completed at least four antenatal care visits. This shortfall, could partially explain Uganda's high maternal mortality ratio of 336 per 100,000 live births (13).

Evaluating to what extent patients are satisfied with ANC services is clinically relevant and of public health significance. Satisfied patients are more likely to comply with treatment, take an active role in their own care, to continue using medical care services and recommend health center's services to others (20).

Recent evidence suggests that women's satisfaction with ANC services remains a critical determinant of continuity of maternal healthcare utilization, particularly in LMICs where health system constraints are persistent.

Although several studies have assessed maternal satisfaction with ANC services in sub-Saharan Africa, evidence from Uganda remains limited and fragmented, especially in urban public health facilities. Existing Ugandan studies have largely focused on ANC attendance and utilization, with less attention given to women's satisfaction and the health system factors associated with it. Furthermore, little is known about the determinants of ANC service satisfaction among pregnant women attending urban public health facilities in Kampala City. Therefore, this study provides context-specific evidence on the level of satisfaction with ANC services and associated factors among pregnant women attending Kawaala Health Centre IV, which may inform strategies aimed at improving quality of maternal healthcare services.

## Materials and methods

### Study design

A cross-sectional analytical study employing quantitative methods of data collection and analysis was conducted.

### Study area

The study was conducted at Kawaala Health Centre IV, located in Lubaga Division, Kampala Capital City Authority (KCCA). The health facility offers a number of maternal health care services such as antenatal and postnatal care, deliveries, family planning, SGBV, STI/STD including HIV testing, counselling and treatment services, immunization, and nutrition services.

Kawaala Health Center IV was chosen for the study as it is located in an urban setting and has a high catchment population. Additionally, Kawaala zone is an area which is predominantly inhabited by people of low socio-economic status who depend mainly on public health care services.

### Target population

The target population were pregnant women attending ANC services at Kawaala Health Centre IV in Kampala city.

### Inclusion and exclusion criteria

#### Inclusion criteria

The study included pregnant women aged between 15–49 years attending ANC services at Kawaala Health Centre IV who consented to participate in the study.

#### Exclusion criteria

The study excluded pregnant women not mentally sound and those who visited the health facility for the first time since they did not know much about the facility.

### Sample size determination

Sample size was determined using the Kish Leslie formula, ($n=\frac{Z^{2}pq}{d^{2}}$) (21).

Where;

*n* = Sample size

*z* = z-value corresponding to a 95% level of significance = 1.96

*p* = Proportion of pregnant women satisfied with ANC services, since we did not know the proportion of pregnant women satisfied with ANC services then we used 50% (*p* = 0.5).

*q* = (1 - p) = (1-0.5) = 0.5

*d* = absolute precision (5%)

Therefore, from the above the sample size was:

$$n=\frac{1.96^{2}\times 0.5\times 0.5}{0.05^{2}}$$

$$n=\frac{0.9604}{0.0025}$$

n=384

Therefore, data was collected from 384 participants.

Participants who did not respond were replaced to ensure having 384 study participants.

### Sampling procedure

A systematic random sampling method was used to select study participants. Pregnant women attending the ANC clinic were issued serial numbers, and the first participant was selected using a lottery method. Thereafter, every third eligible woman was selected for inclusion in the study until the required sample size was achieved.

### Data collection methods and instruments

Data was collected using an interviewer administered structured questionnaire developed for this study. Codes instead of names were used on the questionnaire during interviews with participants to ensure confidentiality and informed consent was sought before proceeding with interview sessions. The interview of respondents on the factors associated with satisfaction with ANC services among pregnant women was conducted in places with no interferences from other people and lasted for approximately 30 to 45 minutes.

### Study variables

#### Dependent variable

Satisfaction with ANC services among pregnant women.

#### Independent variables

Some of the studied factors related to satisfaction with ANC services among pregnant women included age, education level, knowledge of ANC services benefits, proximity to health facility, privacy and confidentiality, attitude of health workers, waiting time, male involvement, health education on ANC, and availability of ANC commodities. Table 1 below shows the study variables with their respective operational definitions, scales of measurement and data collection methods.

**Table 1:** Showing the variables and measurements.

Variable	Operational definition	Scale of measurement	Data collection method
Dependent Variable			
Satisfaction with ANC services	Satisfied with ANC services	Are you satisfied with ANC services at this health facility (1 = Yes, 2 = No) Scale of 1–10 used, score of 7–10(Yes), less than 7 (No)	Interview with pregnant woman
Independent Variables			
Socio-demographic characteristics	Age	Numerical continuous (whole number) and Categorical ordinal (1 = 15–24; 2 = 25–34; 3 = 35–44)	Interview with pregnant woman
	Employment status	Categorical nominal (1= Employed; 2= Unemployed)	Interview with pregnant woman
	Religion	Categorical nominal (1= Catholic; 2= Protestant; 3= Moslem; 4= Anglican 5 = Others-specify)	Interview with pregnant woman
	Marital status	Categorical ordinal (1 = Married; 2= Not married)	Interview with pregnant woman
	Educational level	Categorical ordinal (1= No education, 2 = Primary; 3= Secondary; 3= Tertiary)	Interview with pregnant woman
	Knowledgeable about ANC services	Dichotomous (yes =1 and No-2)	Interview with pregnant woman
Health facility related factors	Availability of user fees	Dichotomous- Do you pay for ANC services (yes =1 and No-2).	Interview with pregnant woman
	Attitude of health workers	Dichotomous- Health workers have positive attitude (yes =1 and No-2).	Interview with pregnant woman.
	Waiting time	Dichotomous- Do you experience long waiting time (30mins) at the health facility? (yes =1 and No-2).	Interview with pregnant woman
	Privacy and confidentiality	Dichotomous- Does the health facility observe privacy and confidentiality? (yes =1 and No-2)	Interview with pregnant woman
	Availability of commodities	Dichotomous- Do you sometimes fail to get drugs or supplies at the health facility when visited? (yes =1 and No-2)	Interview with pregnant woman
	Health talks on ANC	Dichotomous- Does the health facility offer health talks on ANC when visited? (yes =1 and No-2)	Interview with pregnant woman

Satisfaction with ANC services was assessed using a self-rated scale ranging from 1 to 10, where higher scores represented greater satisfaction. Participants scoring between 7 and 10 were classified as satisfied, while those scoring below 7 were categorized as not satisfied. This cut-off has been used in previous patient satisfaction studies and reflects positive perceptions of healthcare services. The questionnaire was developed following extensive literature review and adaptation from previous studies assessing maternal satisfaction with ANC services.

### Quality control methods

#### Reliability of the instrument

The data collection tools were pre-tested before actual data collection to check whether variables of interest come out clearly. The necessary corrections were made to data collection tools before actual data collection. Measurement bias during data collection was avoided by training the research assistants on data collection techniques and tools.

#### Validity of the instrument

A pilot testing was conducted, this included collecting data on small scale to get feedback on whether the instruments of data collection is likely to work in real situations. Content validity was used since it focused on the extent to which the content of an instrument corresponds to the content of the theoretical concept it was designed to measure (22). For an instrument to be accepted as valid, the average index should be 0.7 or above (22).

The content validity index (CVI) was then computed as follows:

$$Content\;Validity\;Index\;(CVI)=\frac{Number\;of\;\;items\;declared\;valid}{Total\;number\;of\;\;items}$$

The CVI of the piloted tool was 0.95.

Checking for completeness and accuracy of completed questionnaires was done on each day of data collection by the researcher.

### Data management and processing

Data checking and cleaning was done simultaneously during data collection. At the end of every day, data was checked for completeness and consistency. Data was backed up by saving in different folders in the computer and on a removable flash disk. Data was exported to STATA 14 for final editing and analysis.

### Bias management

Selection bias was minimized through systematic random sampling of eligible participants attending the ANC clinic. Interviewer bias was minimized by training research assistants on standardized interviewing procedures. To reduce social desirability bias, interviews were conducted privately and participants were assured that their responses would remain confidential and would not affect the services received. Recall bias was minimized by interviewing women immediately after receiving ANC services.

### Data analysis

Data was analyzed using STATA 14. The level of satisfaction with ANC services among pregnant women was presented as percentages (objective one). Socio-demographic data of study participants was presented as frequencies and means. Individual factors and health facility related factors associated with satisfaction with ANC services (objectives two and three). Initially, bivariate analysis was performed between satisfaction with ANC services and independent variables using the Pearson chi-square (*χ*2) test. *P*-values were obtained and a *p*-value <0.05 indicated an association between the independent variable and satisfaction with ANC services. Variables with *p*-values less than 0.20 at bivariate analysis together with clinically and biologically plausible variables identified from literature were considered for inclusion in the multivariable logistic regression model. Statistical significance was set at *p*-value < 0.05 and 95% Confidence Interval (95% CI) was used.

Assumptions for the Pearson Chi-square test, including independence of observations and adequacy of expected cell counts, were assessed before analysis. Prior to fitting the logistic regression model, multicollinearity among independent variables was assessed using the Variance Inflation Factor (VIF). Model fitness was assessed using the Hosmer–Lemeshow goodness-of-fit test. Variables with *p*-values less than 0.05 were considered statistically significant.

## Results

Table 2 above shows the background characteristics of study participants such as their Age (Years), Marital status, Level of Education and Religion. The majority 205 (53.4%) of the study participants were in the age bracket of 24–34 years and only 29 (7.5%) were in the age bracket of 35–44 years. Nearly nine in ten participants (89.3%) were married. More than half 216 (56.3%) of participants had secondary education level and only 5 (1.3%) had informal education. Almost a third 126 (32.8%) of the participants were Catholics, 94 (24.4%) and 81 (21.1%) were Anglicans and Moslem respectively.

**Table 2:** Background characteristics of study participants.

Variable	Category	Number/Frequency	Percentage
Age (Years)	15–24	150	39.1
	25–34	205	53.4
	35–44	29	7.5
Marital status	Single	41	10.7
	Married	343	89.3
Educational level	No Education	5	1.3
	Primary	111	28.9
	Secondary	216	56.3
	Tertiary	52	13.5
Religion	Anglican	94	24.5
	Catholic	126	32.8
	Moslem	81	21.1
	Others (SDA, Orthodox, Pentecostal)	89	21.6
Total Participants		384	100

### The level of satisfaction with ANC services

From the Table 3, more than half 259 (67.4%) of the pregnant women were satisfied with ANC services, and more than a third 140 (36.4%) of the pregnant women in the age bracket 25–34 years were satisfied with the ANC services.

**Table 3:** The level of satisfaction with ANC services.

Age category	Satisfaction with ANC services		Total
	Yes	No	
15–24 years	98 (25.5%)	52 (13.6%)	150 (39.1%)
25–34 years	140 (36.4%)	65 (17.0%)	205 (53.4%)
35–44 years	21 (5.5%)	08 (2.0%)	29 (7.5%)
Total	259 (67.4%)	125 (32.6%)	384 (100%)

### The individual factors associated with satisfaction with ANC services among pregnant women

The individual factors investigated were age, religion, education level, marital status, employment status and knowledge about ANC services. Table 4 below shows the results from the analysis.

**Table 4:** Individual factors associated with satisfaction with antenatal care services among pregnant women.

Independent variable	Category	Satisfaction with ANC services		Total	*χ*2 (df), *p*-value
		Yes	No		
Age	15–24 years	98 (25.5%)	52 (13.6%)	150 (39.1%)	*χ*2 (2) = 0.6978
	25–34 years	140 (36.4%)	65 (17.0%)	205 (53.4%)	
	35–44 years	21 (5.5%)	08 (2.0%)	29 (7.5%)	*P*-value = 0.705
Religion	Catholic	81 (21.1%)	39 (11.7%)	126 (32.8%)	*χ*2 (3) = 0.5621
	Moslem	52 (13.5%)	29 (7.6%)	81 (21.1%)	
	Anglican	65 (16.9%)	29 (7.6%)	94 (24.5%)	*P*-Value =0.905
	Others (SDA, Orthodox and Pentecostal)	61 (15.9%)	28 (5.7%)	89 (21.6%)	
Education level	No Education	4 (1.0%)	1 (0.3%)	5 (1.3%)	*χ*2 (3) = 2.8969
	Primary	76 (19.8%)	35 (9.1%)	111 (28.9%)	
	Secondary	149 (38.9%)	67 (17.4%)	216 (56.3%)	*P*-Value = 0.408
	Tertiary	22 (5.7%)	30 (7.8%)	52 (13.5%)	
Marital status	Married	230 (59.9%)	113 (29.4%)	343 (89.3%)	*χ*2 (1) = 0.2254
	Single	29 (7.5%)	12 (3.2%)	41 (10.7%)	*P*-Value = 0.635
Employment status	Employed	111 (28.9%)	52 (13.5%)	163 (42.5%)	*χ*2 (1) = 0.8647
	Unemployed	148 (38.5%)	73 (19.0%)	221 (57.5%)	*P*-Value = 0.649
Knowledgeable about ANC services	Yes	234 (60.9%)	88 (23.0%)	322 (83.9%)	*χ*2 (1) = 24.7782
	No	25 (6.5%)	37 (9.6%)	62 (16.1%)	*P*-Value < 0.001*

*statistically significant factor, df, degrees of freedom.

Bivariate analysis results using Pearson chi-square (*χ*2) test from Table 4, indicate that being knowledgeable about ANC services is statistically associated with satisfaction with ANC services [*χ*2 = 24.7782; *p* < 0.001] with 60.9% (234/384) were satisfied with ANC services. Factors such as age, religion, educational level, employment and marital status were not statistically associated with satisfaction with ANC services among the pregnant women as shown by the *p*-value (>0.05).

### The health facility factors associated with satisfaction with ANC services

The health facility factors investigated were user fees, attitude of health workers, waiting time, proximity to the health facility, privacy/or confidentiality, male involvement and availability of ANC commodities. Table 5 shows the results from the analysis.

**Table 5:** The health facility factors associated with satisfaction with ANC services.

Independent variable	Category	Satisfaction with ANC services		Total	*χ*2(df), *P*-value
		Yes	No		
User fees	Yes	74 (19.3%)	63 (16.4%)	137 (35.7%)	*χ*2 (1) = 17.5055
	No	185 (48.1%)	62 (16.2%)	247 (64.3%)	*P*-Value <0.001*
Proximity to health facility	<5Kms	86 (22.4%)	31 (8.1%)	117 (30.5%)	*χ*2 (1) = 2.8111
	≥5Kms	173 (45.0%)	94 (24.5%)	267 (69.5%)	*P*-Value = 0.094
Positive attitude of health workers	Yes	236 (61.5%)	83 (21.6%)	319 (83.1%)	*χ*2 (1) = 36.6373
	No	23 (5.9%)	42 (11.0%)	65 (16.9%)	*P*-Value <0.001*
Waiting time	<1hr	249 (64.8%)	91 (23.7%)	340 (88.5%)	*χ*2 (1) = 45.2662
	≥1hr	10 (2.6%)	34 (8.9%)	44 (11.5%)	*P*-Value <0.001*
Privacy/confidentiality	Yes	257 (66.9%)	120 (31.3%)	377 (98.2%)	*χ*2 (1) = 4.9082
	No	2 (0.5%)	5 (1.3%)	7 (1.8%)	*P*-Value =0.027*
Availability of ANC commodities	Yes	67 (17.4%)	16 (4.2%)	83 (21.6%)	*χ*2 (1) = 8.4989
	No	192 (50.0%)	109 (28.4%)	301 (78.4%)	*P*-Value = 0.004*
Male partner involvement	Yes	235 (61.2%)	102 (26.6%)	337 (87.8%)	*χ*2 (1) = 6.5478
	No	24 (6.2%)	23 (6.0%)	47 (12.2%)	*P*-Value = 0.011*

*statistically significant factor; (df)=degrees of freedom.

From Table 5 above, user fees was statistically significantly associated with satisfaction with ANC services [*χ*2 = 17.5055; *p* < 0.001] with 74.9% (185/247) of the pregnant women who never report user fees were satisfied with ANC services.

Positive attitude of health workers is statistically significantly associated with satisfaction with ANC services [*χ*2 = 36.6373; *p* < 0.001] with 74.0% (236/319) of the pregnant women who reported positive attitudes of health workers were satisfied with ANC services.

Waiting time at health facility is statistically significantly associated with satisfaction with ANC services [*χ*2 = 45.2662; *p* < 0.001] with 73.2% (249/340) of the pregnant women who reported waiting of less than an hour were satisfied with ANC services.

Privacy/or confidentiality is statistically significantly associated with satisfaction with ANC services [*χ*2 = 4.9082; *p* = 0.027] with 68.2% (257/377) of the pregnant women who reported privacy/or confidentiality were satisfied with services.

Availability of ANC commodities is statistically significantly associated with satisfaction with ANC services [*χ*2 = 8.4989; *p* = 0.004] with 80.7% (67/83) of the pregnant women who reported availability of ANC commodities during ANC visits were satisfied with the services.

Male involvement in ANC visits is statistically significantly associated with satisfaction with ANC services [*χ*2 = 6.5478; *p* = 0.011] with 69.7% (235/337) of the pregnant women who reported male involvement during ANC visits were satisfied with the services.

Proximity to health facility is not statistically associated with satisfaction with ANC services as indicated by the *p*-value (>0.05).

### Factors independently associated with satisfaction with ANC services

The variables carried forward into the logistic regression model were knowledge about ANC services, health workers’ attitude, waiting time, privacy, user fees, and male involvement that were found to be statistically significant at bivariate analysis.

The results of the model used are shown in Table 6. The logistic regression model that best predicts satisfaction with ANC services from the various predictors and has *p*-value **<0.001.** Prior to multivariable analysis, multicollinearity among predictor variables was assessed and no evidence of multicollinearity was observed (all VIF values were less than 10). The logistic regression model demonstrated adequate fit according to the Hosmer–Lemeshow goodness-of-fit test (*p* > 0.05).

**Table 6:** Odds ratios and *P*-values obtained from the logistic regression model.

Independent variable	Crude Odds Ratio	*P*-Value	95% CI	Adjusted Odds Ratio	*P*-Value	95% CI
Knowledgeable about ANC services						
Yes	3.9	<0.001*	2.24–6.91	4.2	**<0**.001*	2.23–7.81
No	1.0			1.0		
Waiting time						
<1hr	3.2	<0.001*	1.66–5.99	2.8	**0**.009*	1.30–5.99
≥1hr	1.0			1.0		
Privacy/confidentiality						
Yes	5.3	0.047*	1.02–27.99	3.3	0.230	0.47–22.24
No	1.0			1.0		
Positive attitude of health workers						
Yes	5.6	<0.001*	3.19–10.01	5.9	**<0**.001*	3.13–11.25
No	1.0			1.0		
Availability of ANC commodities						
Yes	2.2	0.009*	1.21–3.89	3.0	**0**.002*	1.53–6.06
No	1.0			1.0		
User fees						
No	1.5	0.057	0.99–2.38	1.5	0.110	0.91–2.52
Yes	1.0			1.0		
Male involvement						
Yes	2.2	0.012*	1.19–4.09	1.7	0.158	0.82–3.38
No	1.0			1.0		

*statistically significant factor.

From Table 6 above, the pregnant women who were knowledgeable about ANC services were 4.2 times more likely to be satisfied with ANC services than those who were not knowledgeable and this was statistically significant [AOR=4.2: 95% CI 2.23–7.81: *P* < 0.001].

The pregnant women who waited less than an hour during attendance of ANC services were 2.9 times more likely to be satisfied with ANC services than those who waited more than an hour and this was statistically significant [AOR=2.8: 95% CI 1.30–5.99: *P* = 0.009].

The pregnant women who reported positive attitude from health workers during attendance of ANC services were 5.9 times more likely to be satisfied with ANC services than those who reported negative attitude and this was statistically significant [AOR=5.9: 95% CI 3.13– 11.25: *P* < 0.001].

The pregnant women who reported availability of ANC commodities during attendance of ANC services were 3.0 times more likely to be satisfied with ANC services than those who reported lack of ANC commodities and this was statistically significant [AOR=3.0: 95% CI 1.53–6.06: *P* = 0.002].

In summary, satisfaction with ANC services is associated with knowledge about ANC services, waiting time, attitude of health workers, and availability of ANC commodities. Satisfaction with ANC services is not associated with age, level of education, religion, marital status, socio-economic status, user fees, distance from health facility, privacy/confidentiality and male involvement.

## Discussion of results

### Proportion of pregnant women satisfied with antenatal care services

It has been observed that more than half 259 (67.4%) of the pregnant women were satisfied with ANC services at Kawaala HC IV in Lubaga Division, Kampala city. This finding is in agreement with a study conducted on satisfaction with focused antenatal care service and associated factors among pregnant women which reported that more than half of the respondents (60.4%) were satisfied with the ANC services (23). In another study on pregnant women's satisfaction and associated factors, 70.3% of the pregnant women were satisfied with antenatal care services (24).

The probable reason for the high satisfaction with ANC services among the pregnant women in this study could be due to positive attitudes of the health workers, proximity to health facility and high literacy level on ANC services. This is supported by several studies which reported that women who travel less than 10 km to the ANC clinics were satisfied with the ANC services and most of them attended at least 4 ANC visits (25, 26).

Additionally, this may be because the physical accessibility of health facilities in urban areas with lesser transportation costs involved could encourage pregnant mothers to have access to and utilize the services. Better access to information of the urban community concerning the benefit of ANC services could be also contributing to the high satisfaction with the services.

### The individual factors associated with satisfaction with ANC services

#### Knowledge about ANC services

Being knowledgeable about services offered during antenatal care is associated with satisfaction with ANC services. The study findings indicate that adolescents who were knowledgeable about ANC services had higher odds of satisfaction compared to those who did not have knowledge about ANC services. Women who are knowledgeable about ANC services may have realistic expectations regarding the care provided, are more likely to actively participate during consultations, and can better appreciate the benefits of the services received, thereby enhancing satisfaction.

This agrees with earlier studies (25, 26), which reported that women who had good knowledge about ANC were satisfied with the services and attended four or more visits compared to those with poor knowledge. In addition, a Ugandan study reported that lack of knowledge about the benefits of ANC services was associated with dissatisfaction with ANC services among pregnant women and this consequently affected attendance of at least 4 ANC visits (27).

The probable reason for this finding could be that having knowledge about ANC services empowers pregnant women to demand for services responsive to their needs hence subsequently leading to satisfaction.

Another probable reason for knowledge about ANC services being associated with satisfaction with ANC services could be that pregnant mothers who are knowledgeable are likely to give constructive feedbacks after the services with the aim of improving the quality of services through taking actions to address the identified gaps.

### The health facility factors associated with satisfaction with ANC services

#### Waiting time

The waiting time at health facility is significantly associated with satisfaction with ANC services among the pregnant women. The study finding indicated that pregnant women who wait less than one hour to get ANC services are more likely to be satisfied with the service compared to those who wait for more than an hour. Shorter waiting times may improve satisfaction because women perceive the services as efficient and responsive to their needs. Long waiting periods may increase fatigue, inconvenience, and dissatisfaction with service delivery. This agrees with various studies which reported that women who reported less waiting time at the health facility were 2.9 times more likely to be satisfied with ANC services than those who stay long at the health facility (28). This finding also agrees with study which reported that clients whose waiting time was less than an hour were 2 times likely to be satisfied as compared to those whose waiting times were greater than an hour (24).

The probable reason for this finding could be that less waiting time is associated with getting timely services and hence the pregnant mothers will perceive it that their time is not being wasted hence leading to satisfaction with ANC services.

#### Attitude of health workers

The study finding indicate that positive attitude of the health workers was associated with satisfaction with ANC services among pregnant women attending ANC. Respectful and empathetic interactions foster trust between clients and healthcare providers, which positively influences women's perceptions of service quality and overall satisfaction. This agrees with earlier studies (2, 29), which reported that health workers’ positive attitude at the ANC points of care was associated with satisfaction with ANC services among the pregnant women.

Additionally, the study findings agree with studies (20, 30), one of important determinant of mothers’ satisfaction with antenatal services in developing countries is interpersonal behavior and in these studies mothers were satisfied if they were greeted and spoken to politely.

The probable reason for the study finding is that good provider-patient relationships are therapeutic and is the most important component of good medical practice, because not only it identifies problems quickly and clearly, but it also defines expectation and helps establish trust and rapport between the health worker and patient.

In addition, positive attitudes of health workers encompass empathy, which is crucial to the effective achievement of patient satisfaction with ANC services because it encapsulates sensitivity to both the informational and emotional aspects of communication (33).

#### Availability of ANC commodities

The study has revealed that availability of ANC commodities is statistically significantly associated with satisfaction with ANC services among pregnant women. Availability of essential medicines and supplies may enhance satisfaction by ensuring that women receive comprehensive care without incurring additional out-of-pocket expenses. This agrees with an earlier Ugandan study (31), which reported that the availability of drugs and supplies was independently associated with satisfaction with ANC services. In the same study, lower satisfaction with ANC services was due to shortage of supplies and stocking out of essential drugs.

This finding also agrees with another cross-sectional study conducted in Nigeria on ANC satisfaction where findings showed that wide spread and major deficiencies in the availability of essential equipment and drugs was associated with women's satisfaction with ANC (32).

The probable reason for this finding is because once pregnant women are able to get all the drugs and supplies during each ANC visits, they will appreciate the services rendered to them and hence be satisfied with it.

Additionally, in Uganda, the government funds provision of services in public health facilities, therefore patients expect to get free services. However, in a number of instances, unprepared pregnant women are required to buy commodities and supplies out of stock against their expectations, which may lead to poor satisfaction levels.

In the present study, age, educational level, religion, and marital status were not significantly associated with ANC satisfaction. Similar findings have been reported in other studies suggesting that perceptions of service quality may be influenced more by health system factors than by socio-demographic characteristics.

## Conclusion

Women's satisfaction with ANC services at Kawaala Health Centre IV was influenced predominantly by modifiable health system factors, including waiting time, provider attitude, availability of ANC commodities, and knowledge about ANC services. Interventions aimed at strengthening health education, improving provider-client interactions, reducing waiting times, and ensuring continuous availability of essential commodities may enhance client satisfaction and potentially improve utilization and continuity of ANC services.

### Limitations of the study

This study had several limitations. First, the cross-sectional design precludes causal inference between the identified factors and satisfaction. Second, being conducted at a single health facility limits the generalizability of findings to other settings. Third, satisfaction was measured through self-report and may have been affected by social desirability bias. Fourth, the questionnaire used was not based on a standardized validated satisfaction tool. Finally, women who did not attend ANC services were excluded, potentially limiting understanding of satisfaction among non-users of ANC services. The study did not assess important obstetric and socioeconomic variables such as parity, gravidity, gestational age, number of previous ANC visits, and household income, which may influence satisfaction with ANC services.

### Recommendations

The health facility should provide routine health talks about ANC services as this would help to improve the health literacy of the pregnant women. This empowers pregnant women to be able to appraise the services being offered to them and subsequently provide feedback on the quality of ANC services.

The District Health Officer (DHO) and Ministry of Health (MOH) should ensure that health facilities have adequate staff to eliminate long waiting hours.

The health facility should put in place measures to reduce unnecessary delays to receive ANC services through teamwork and multi-tasking to avoid long queues at the health facilities.

The health facility and MOH should ensure health workers are trained on customer care to improve their attitude and handling of pregnant women during ANC visits as positive attitude from health workers is associated with satisfaction with the services.

The health facilities and MOH should ensure availability of ANC commodities at all times through proper forecasting and quotation as well as timely submission of bimonthly drug/supplies orders.

The health facilities should also be trained on supply chain management to avoid unnecessary commodities stock outs due to poor supply chain management.

Another study should be done using mixed methods of research and also collect data from the community not only in health facility.

## Data Availability

The original contributions presented in the study are included in the article/Supplementary Material, further inquiries can be directed to the corresponding author.
